# Emerging Roles of Post-Translational Modifications in Nucleotide Excision Repair

**DOI:** 10.3390/cells9061466

**Published:** 2020-06-15

**Authors:** Barbara N. Borsos, Hajnalka Majoros, Tibor Pankotai

**Affiliations:** Department of Oral Biology and Experimental Dental Research, Faculty of Dentistry, University of Szeged, 83 Tisza L. krt., H-6722 Szeged, Hungary; borsos.barbara.nikolett@gmail.com (B.N.B.); majoroshajnalka@gmail.com (H.M.)

**Keywords:** TC-NER, GG-NER, E3 ligases, DUBs, ubiquitylation, K63 chains, K48 chains

## Abstract

Nucleotide excision repair (NER) is a versatile DNA repair pathway which can be activated in response to a broad spectrum of UV-induced DNA damage, such as bulky adducts, including cyclobutane-pyrimidine dimers (CPDs) and 6–4 photoproducts (6–4PPs). Based on the genomic position of the lesion, two sub-pathways can be defined: (I) global genomic NER (GG-NER), involved in the ablation of damage throughout the whole genome regardless of the transcription activity of the damaged DNA locus, and (II) transcription-coupled NER (TC-NER), activated at DNA regions where RNAPII-mediated transcription takes place. These processes are tightly regulated by coordinated mechanisms, including post-translational modifications (PTMs). The fine-tuning modulation of the balance between the proteins, responsible for PTMs, is essential to maintain genome integrity and to prevent tumorigenesis. In this review, apart from the other substantial PTMs (SUMOylation, PARylation) related to NER, we principally focus on reversible ubiquitylation, which involves E3 ubiquitin ligase and deubiquitylase (DUB) enzymes responsible for the spatiotemporally precise regulation of NER.

## 1. Introduction

In eukaryotic cells exposed to UV-irradiation, two different modes of nucleotide excision repair (NER) are activated: (I) global genomic-nucleotide excision repair (GG-NER) and (II) transcription-coupled-nucleotide excision repair (TC-NER), which is involved in the recognition of distorted DNA and determines both spatial and time-related preferences [1,2]. NER contributes to the elimination of helix-distorting lesions, including cyclobutane-pyrimidine dimers (CPDs), 6-4 photoproducts (6-4PPs), and other bulky adducts; hence, it maintains genome stability. The complexity of NER pathways, the size and scaffold structure of the lesion, and the functions of the repair factors need to be orchestrated in a multi-stage process. The coordinated interactions of more than 30 proteins involved in NER are mainly controlled by various post-translational modifications (PTMs), including ubiquitylation, ADP-ribosylation (PARylation), and SUMOylation.

Ubiquitylation is catalyzed by E1 activating, E2 conjugating, and E3 ligase enzymes [3]. During ubiquitylation, one or more ubiquitin molecule(s) (referred to as monoubiquitylation and polyubiquitylation, respectively) can be transferred to the target protein, and this process results in different outcomes regarding the type of ubiquitin linkage. For instance, in most cases, K48-linked polyubiquitylation leads to the proteasomal degradation of the target protein, while K63-linked chains take part in other cellular processes [4]. During ubiquitylation, it is essential to maintain the balance between the E3 ligases and deubiquitylases (DUBs) responsible for the removal of the ubiquityl groups. CRL4^DDB2^ and CRL4^CSA^ (Cullin-RING ubiquitin ligase) E3 ligase complexes catalyze the initial steps, determining the following downstream steps in GG-NER and TC-NER, respectively. In GG-NER, XPC (Xeroderma pigmentosum, complementation group C), which is one of the first sensors of DNA damage, can be either polyubiquitylated at K48-linked chains by CRL4^DDB2^ or at K63-linked chains by RNF111 (Ring Finger Protein 111, also known as Arkadia), resulting in different outcomes. These sites can be deubiquitylated by UBP12 (Ubiquitin specific protease 12) and USP24 (Ubiquitin specific peptidase 24) (at K48) as well as by USP7 (Ubiquitin specific peptidase 7) and OTUD4 (OTU deubiquitinase 4) (at K63). In TC-NER, CSB (Cockayne syndrome B protein), which traverses together with the RNAPII (RNA polymerase II), should be removed from the DNA by CRL4^CSA^- and BRCA1-BARD1 (Breast Cancer Type 1 Susceptibility Protein - BRCA1 Associated RING Domain 1)-mediated ubiquitylation-coupled proteasomal degradation to allow access for further repair proteins. However, as long as necessary, CSB is deubiquitylated by USP7 at these sites to remain recruited at the lesions. In certain cases, the elongating RNAPII (S2P RNAPII) should be removed from the damage site. This process involves E3 ligases, including BRCA1-BARD1, NEDD4 (Neural Precursor Cell Expressed, Developmentally Down-Regulated 4), and Elongin A/B/C-Cul5-RBX2 (RING-box protein 2) complexes [5,6,7]. These ubiquitylation steps can be reversed by UBP2 (Ubiquitin specific protease 2) and UBP3 (Ubiquitin specific protease 3) [8].

PARylation is also a reversible PTM, which is catalyzed by poly(ADP-ribose) polymerases (PARPs), responsible for the formation of an ester bond between ADP-ribose and the carboxyl-group of acidic amino acids [9,10]. Among these enzymes, PARP-1 (Poly(ADP-ribose) polymerase 1) has been shown to be involved in both single- and double-strand break repair [11]. Interestingly, while the depletion of PARP-1 results in failure of DNA damage repair processes, it has a controversial effect on mitochondrial activity. However, less information has been ascertained about the negative impact of PARP-1 on mitochondrial biogenesis, which has to be further elucidated [12,13]. Moreover, since PARylation is a reversible process, PAR chains can be hydrolyzed by PAR glycohydrolase (PARG) [14].

SUMO enzymes are produced in the cells as inactive pro-enzymes and they are first activated by sentrin/small ubiquitin-like modifier-specific proteases (SENPs), which are also capable of catalyzing the reversal deSUMOylation process [15]. Similar to ubiquitylation, SUMO conjugation requires E1 activating, E2 conjugating, and E3 ligase enzymes. The concerted cooperation of these enzymes leads to the formation of an iso-peptide bond between the C-terminal glycine residue of SUMO and the lysine amino acid of the target protein [16]. In humans, four known SUMOs are present: SUMO-1, SUMO-2, SUMO-3, and SUMO-4. Additionally, it is important to note that RNF111, a SUMO-targeted ubiquitin ligase (STUbL), plays a pivotal role during NER by triggering important crosstalk between SUMOylation and ubiquitylation [17].

In this review we introduce the most important PTMs that play an essential role in NER, although we principally emphasize the regulatory role of ubiquitylation during GG-NER and TC-NER. Furthermore, we highlight the opposing roles of these PTMs in the same target protein. In turn, the balance of these actions ensures the coordination of the repair pathway. These processes jointly contribute to the preservation of genome integrity, which may prevent cancerous malformations.

## 2. Ubiquitylation-Mediated Processes During Nucleotide Excision Repair

### 2.1. Global Genomic-Nucleotide Excision Repair

**Figure 1:**
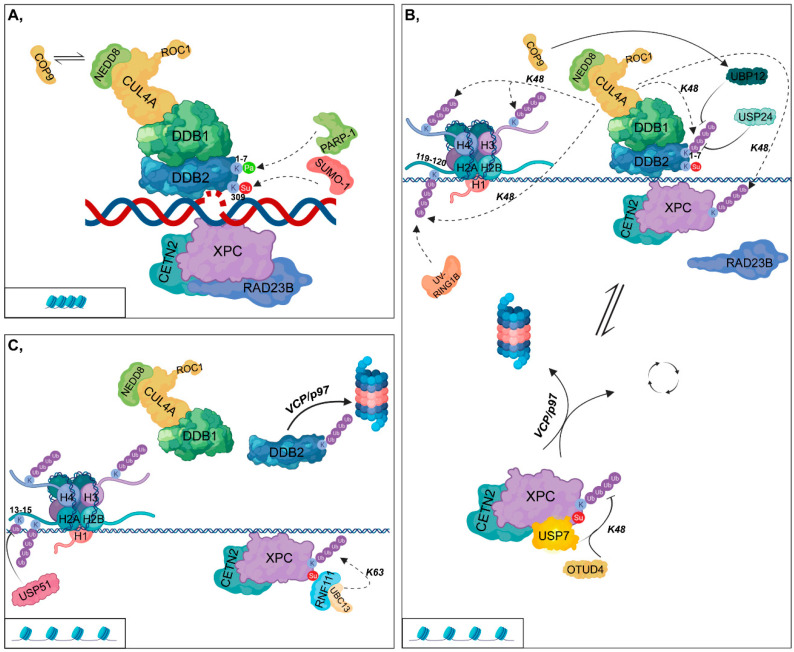
Global genomic-nucleotide excision repair (**A**) XPC (Xeroderma pigmentosum, complementation group C), complexed with CETN2 (Centrin 2) and RAD23B (RAD23 Homolog B), binds to the opposite strand of helix-distorted lesions. In contrast, for CPDs (cyclobutane-pyrimidine dimers), XPC only binds to the damaged regions after the recruitment of the DDB1-DDB2 (DNA-Damage-Binding Protein 1 - DNA-Damage-Binding Protein 2) complex, which then forms a larger complex (CRL4^DDB2^) with CUL4A (Cullin 4A) and ROC1 (RING-box protein 1/RING subunit of SCF). SUMO-1 (Small ubiquitin-related modifier 1) and PARP-1 (Poly(ADP-ribose) polymerase 1) catalyze the SUMOylation and PARylation of DDB2 at K309 and at K1-7, respectively. CRL4^DDB2^ activation requires the binding of NEDD8 (Neural Precursor Cell Expressed, Developmentally Down-Regulated 8). However, under physiological conditions, COP9 (Constitutive photomorphogenesis 9) replaces NEDD8, leading to the inactivation of the complex. (**B**) Following DNA-binding, RAD23B dissociates from XPC and at the same time, XPC is polyubiquitylated. CUL4A can differentially affect DDB2 and XPC, although it catalyzes the K48-linked polyubiquitylation of both proteins. UBP12 (Ubiquitin specific protease 12) (recruited by COP9) and USP24 (Ubiquitin specific peptidase 24) remove the ubiquityl groups from DDB2. The CRL4^DDB2^ complex catalyzes the K48-linked polyubiquitylation of H3, H4, and H2A at K119/120, while UV-RING1B (UV-Ring Finger Protein 1) polyubiquitylates H2A at K119. OTUD4 (OTU deubiquitinase 4) and USP7 (Ubiquitin specific peptidase 7) participate in the removal of K48-linked ubiquityl groups from XPC. According to the ubiquitylation state of XPC, it can be either recycled or degraded in the proteasome. (**C**) USP51 (Ubiquitin specific peptidase 51) removes the ubiquityl groups from H2A K13/15. RNF111 (Ring Finger Protein 111, also known as Arkadia)-UBC13 (Ubiquitin-conjugating enzyme E2 13) catalyzes the K63-linked non-proteolytic polyubiquitylation of XPC. The K48-linked polyubiquitylation of DDB2 results in its dissociation from the CRL4^DDB2^ complex and its subsequent VCP/p97 (Valosin-containing protein)-mediated proteasomal degradation. Chromatin structural changes (from compacted (**A**) to a more relaxed form (**B**,**C**)) are represented in rectangles.

In the first step of GG-NER, XPC in the complex with RAD23B (RAD23 Homolog B) and CETN2 (Centrin 2) can directly bind to the opposite DNA strand, where helix-distorting lesions have accumulated [18,19]. In contrast, CPDs slightly distort the DNA; therefore, XPC is recruited to such damaged sites only after the binding of the UV-DDB (UV-damaged DNA-binding protein) complex [20]. This complex comprises DDB1 (DNA-Damage-Binding Protein 1) and DDB2 (DNA-Damage-Binding Protein 2), which then forms a larger complex (CRL4^DDB2^) with CRL composed of CUL4A (Cullin 4A) E3 ubiquitin ligase and RBX1/ROC1 (RING-box protein 1/RING subunit of SCF) E2 ubiquitin-conjugating enzyme [21]. DDB1 ensures the linkage between DDB2 and the CRL complex, while DDB2, which is the DNA-binding component, is required for recruiting XPC to CPDs by facilitating the looping of the broken DNA strand [22] (Figure 1A).

This process is regulated by PTMs of DDB2 as follows: (I) SUMOylation at K309 by SUMO-1 (Small ubiquitin-related modifier 1), (II) PARylation by PARP-1 and (III) ubiquitylation by UV-DDB itself [23,24]. SUMOylation of DDB2 ensures the binding of XPC to the kinked DNA region [23] (Figure 1A,B). However, PARylation and ubiquitylation exert opposite effects on DDB2; they modify K1-7 located within the first 40 N-terminal amino acid residues as follows: (I) PARylation results in the stabilization of the protein and promotes the recruitment of ALC1 (Amplified in liver cancer 1) chromatin remodeler; (II) K48-linked polyubiquitylation leads to its VCP/p97 (Valosin-containing protein)-mediated proteasomal degradation [24,25] (Figure 1A,C). USP24 DUB is capable of removing the ubiquityl groups from DDB2 and therefore contributes to the fine-tuning of GG-NER [26] (Figure 1B).

Activation of the CRL4^DDB2^ complex requires its interaction with NEDD8 (Neural Precursor Cell Expressed, Developmentally Down-Regulated 8). Under physiological conditions, the binding of the COP9 (Constitutive photomorphogenesis 9) signalosome (CSN) catalyzes the removal of NEDD8 from CUL4 to inactivate CRL4^DDB2^ [27]. After UV-irradiation, COP9 dissociates from the complex, allowing its neddylation and subsequent activation [21] (Figure 1A). Additionally, COP9 also has DUB activity and in fission yeast, it can recruit UBP12 to the CRL4^DDB2^ complex by which K48-linked ubiquityl groups are removed from the complex [28] (Figure 1B).

As noted above, RAD23B plays an indispensable role in facilitating the binding of XPC to helix-distorting lesions generated by UV-irradiation. Next, interaction ceases between the two proteins and simultaneously, XPC is ubiquitylated [29]. However, polyubiquitylation of XPC can result in different outcomes depending on the type of ubiquitin linkage (K48 or K63). Moreover, DDB2 and RNF111 are essential for GG-NER, although they affect XPC differently: (I) DDB2 presumably catalyzes the K48-linked polyubiquitylation of XPC, and thus leads to its increased DNA-binding affinity; (II) while UBC13 (Ubiquitin-conjugating enzyme E2 13)/RNF111 mediates the non-proteolytic K63-linked polyubiquitylation of XPC, which results in the dissociation of XPC from DNA [17,30,31]. This step is reversible, since human DUB enzymes, USP7 and OTUD4 have been shown to catalyze the removal of ubiquityl groups from XPC probably by trimming K48-linked ubiquitin chains to permit RNF111-related K63-linked polyubiquitylation of XPC at the same site (Figure 1B,C) [32,33]. This reaction removes XPC from the DNA and is therefore crucial for the later recruitment of XPF-ERCC1 (Xeroderma pigmentosum, complementation group F - Excision Repair Cross-Complementation Group 1) and XPG (Xeroderma pigmentosum, complementation group G) to the appropriate position [34]. Although the removal of these polyubiquitin chains by USP7 leads to the unmodified state of XPC, it does not contribute to its proteasomal degradation but rather facilitates the recycling of the protein. In contrast, in the absence of USP7, XPC is targeted for VCP/p97-mediated proteasomal degradation [32] (Figure 1B). 

CRL4^DDB2^ can ubiquitylate H3 and H4 histones as well as H2A at its K119/K120 residues at damaged DNA sites. This leads to chromatin decondensation and gives NER factors access to the damaged site [35,36,37]. CRL4^DDB2^ catalyzes the K48-linked polyubiquitylation of these histones as well [22]. Furthermore, an additional E3 ligase complex, UV-RING1B (UV-Ring Finger Protein 1), specifically ubiquitylates H2A at K119. This results in the recruitment of ZRF1 (Zuotin-related factor 1) and then leads to the reassembly of several multiprotein complexes [38,39,40] (Figure 1B). Although the deubiquitylation events on H2A have not yet been entirely clarified, it has been recently shown that USP51 (Ubiquitin specific peptidase 51) plays an essential role in deubiquitylation at K13–15 residues of H2A [41] (Figure 1C).

### 2.2. Transcription-Coupled-Nucleotide Excision Repair

**Figure 2:**
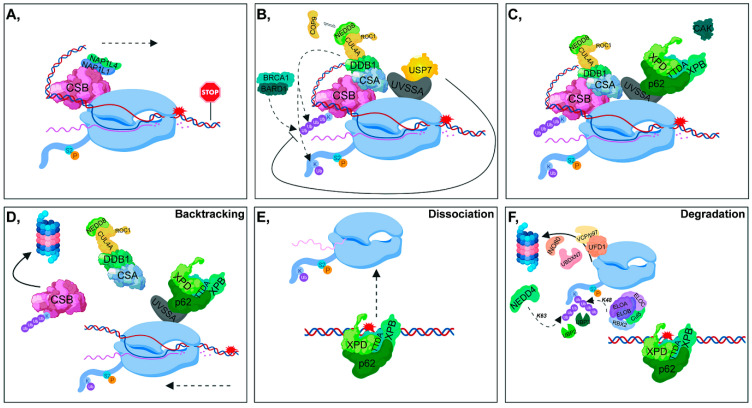
Transcription-coupled-nucleotide excision repair (**A**) Following UV damage, elongating RNAPII (S2P RNAPII) is stalled, then NAP1L1 (Nucleosome assembly protein 1-like 1) as well as NAP1L4 (Nucleosome assembly protein 1-like 4) bind to CSB (Cockayne syndrome B protein). This contributes to the unwinding of the DNA and the forward translocation of RNAPII (indicated with a dashed arrow). (**B**) CRL4^CSA^ (composed of CUL4A, ROC1 - RING-box protein 1/RING subunit of SCF -, DDB1, and CSA - Cockayne syndrome A protein -) binds to and ubiquitylates CSB, taking part in the subsequent removal of CSB. CRL4^CSA^ ubiquitylates RNAPII. Additionally, CSB can also be ubiquitylated by the BRCA1-BARD1 (Breast Cancer Type 1 Susceptibility Protein - BRCA1 Associated RING Domain 1) complex. In contrast, UVSSA (UV-stimulated scaffold protein A), by recruiting USP7 DUB is involved in the deubiquitylation and subsequent stabilization of CSB. (**C**) Next, USP7 dissociates from UVSSA allowing the binding of TFIIH (through its p62 subunit) to UVSSA. (**D**) Following the removal of USP7, CSB is ubiquitylated and degraded by the 26S proteasome. TFIIH is responsible for the reverse translocation of RNAPII (indicated with a dashed arrow). (**E**) Dissociation of RNAPII from DNA. (**F**) In the ubiquitin-related proteasomal degradation of RNAPII, NEDD4 (Neural Precursor Cell Expressed, Developmentally Down-Regulated 4) (K63-linked) and the Elongin A/B/C-Cul5-RBX2 (RING-box protein 2) complex (K48-linked) are involved. UBP2 (Ubiquitin specific protease 2) participates in the deubiquitylation of K63-linked, while UBP3 (Ubiquitin specific protease 3) takes part in the removal of K48-linked polyubiquitin chains from RNAPII. Additionally, the chromatin remodeler INO80, the segregase VCP/p97, and its co-factors, UFD1 (Ubiquitin fusion degradation protein 1) and UBDXN7, are also involved in the proteasomal degradation of RNAPII.

One of the most tenacious activation signals for TC-NER is stalled S2P RNAPII, which covers an approximately 30 nucleotide DNA region upstream of the damaged site, preventing proper access of repair proteins to the lesion. Under physiological conditions, CSB interacts with the S2P RNAPII, and this binding is further enhanced following UV-induced DNA damage. The ATP-dependent chromatin remodeling activity of CSB can be stimulated by the binding of NAP1L1 (Nucleosome assembly protein 1-like 1) and NAP1L4 (Nucleosome assembly protein 1-like 4) histone chaperones to CSB [42]. Subsequent recruitment of CSB to bulky lesions results in DNA bending and generates a more favorable conformation to which downstream repair factors can bind [43] (Figure 2A). In addition, CSB and CSA (Cockayne syndrome A protein) jointly contribute to the recruitment of factors involved in chromatin remodeling, such as the nucleosome binding protein HMGN1 (High-Mobility Group Nucleosome Binding 1), p300 histone acetyl-transferase and the pre-mRNA splicing involved protein XAB2 (XPA Binding Protein 2) [43].

Since both CSA and DDB2 (involved in TC-NER and in GG-NER, respectively) are part of the DCAF (DDB1- and CUL4-associated factors) complex, both can form a complex with CUL4A E3 ubiquitin ligase (CRL4^CSA^ and CRL4^DDB2^, respectively) [21]. The ubiquitin ligase activity of CRL4^CSA^ is also coordinated by the binding of COP9: (I) under physiological conditions, it strongly inhibits the ligase activity of CRL4^CSA^, (II) in response to DNA damage, it is released from CRL4^CSA^ and thus promotes the E3 ligase activity of the complex [21,44]. In contrast to CRL4^DDB2^, COP9 does not dissociate from the CRL4^CSA^ complex immediately after UV-irradiation [21] (Figure 2B).

To promote the access of NER factors to the lesion, in most cases, RNAPII needs to be translocated from the site of DNA damage. In this process, a key function is attributed to CSB, the presence of which at the RNAPII-stalled region is regulated by UVSSA (UV-stimulated scaffold protein A) and CSA E3 ligase as well as USP7 DUB. CSA and UVSSA have opposing effects on CSB: (I) CSA (through the CRL4 complex) ubiquitylates CSB, and contributes to its removal through its ubiquitin-binding domain (UBD), (II) UVSSA stabilizes CSB by recruiting USP7. In addition, BRCA1-BARD1 is also involved in the ubiquitin-mediated removal of CSB [5] (Figure 2B). Dissociation of CSB is a crucial step in lesion removal, since as long as CSB is present at the damage site, the incision step cannot be fulfilled [45] (Figure 2D). In contrast, it has been demonstrated that CSB can only be ubiquitylated when the repair process has been completed, which facilitates transcription restart [44].

The p62 subunit of TFIIH binds to DNA through UVSSA, presumably at the same site occupied by USP7 [46,47]. Consequently, USP7 dissociates from UVSSA, leading to the degradation of CSB through a ubiquitin-dependent proteasomal manner, promoting the further incision steps of TC-NER [47,48] (Figure 2C,D). Polyubiquitylation of UVSSA at K414 prevents its binding to USP7 and marks it for proteasomal degradation resulting in the malfunction of TC-NER [48,49].

The fate of RNAPII varies according to the type of DNA damage: (I) lesion bypass (Figure 2A), (II) backtracking (Figure 2D), (III) dissociation from DNA (Figure 2E), (IV) ubiquitin-related proteasomal degradation of RNAPII, which is the so-called ‘last resort’ mechanism (Figure 2F) [50,51]. CSB and TFIIH play a pivotal role in the displacement of RNAPII, although they have opposite effects according to the orientation of translocation: CSB and TFIIH possess forward and reverse translocase activities, respectively. At minor lesions, CSB contributes to the resumption of transcription elongation by pulling the DNA strand [52] (Figure 2A). However, at bulky lesions, the reverse translocase activity of TFIIH predominates [53] (Figure 2D). 

In the ‘last resort’ pathway, when RNAPII has to be completely removed from DNA, RPB1 (the largest subunit of RNAPII) is ubiquitylated by several E3-ligases. NEDD4 catalyzes K63-linked polyubiquitylation of RNAPII, of which the ubiquitin chain can be shortened by UBP2. Subsequently, the remaining monoubiquityl group is processed to K48-linked polyubiquityl chains by the Elongin A/B/C-Cul5-RBX2 complex, resulting in the proteasomal degradation of the S2P RNAPII. This step can be reversed by the contribution of UBP3 [6,7]. The chromatin remodeler INO80, the segregase VCP/p97, and its co-factors, UFD1 (Ubiquitin fusion degradation protein 1) and UBDXN7 are also involved in the degradation process [54] (Figure 2F).

Following lesion removal, several factors mediate the resumption of transcription: (I) the FACT (Facilitates chromatin transcription) complex ensures the turnover of H2A and H2B from the damaged site, and therefore destabilizes the nucleosomes, (II) DOT1L (DOT1 like histone lysine methyltransferase) catalyzes the dimethylation of H3K79, hence accelerating the transcription process, (III) HIRA (Histone regulator A) facilitates the deposition of H3.3 at the lesions, and since H3.3 is mainly present at transcriptionally active regions, the accumulation of this histone variant promotes the recovery of RNA synthesis [55,56,57]. Furthermore, when lesion removal has been completed, CAF1 (Chromatin Assembly Factor 1) and ASF1 (Anti-silencing Factor 1) recruit the H3.1 histone variant to the DNA [58,59,60]. By reorganizing the chromatin structure, these factors strongly contribute to the proper restart of transcription.

### 2.3. DNA Damage Verification and Repair During NER

Binding and subsequent removal of XPC and backtracking or displacement of RNAPII from the damaged site contribute to the recruitment of downstream factors in GG-NER and TC-NER, respectively. At this stage, the two sub-pathways merge and the subsequent steps will be common.

**Figure 3:**
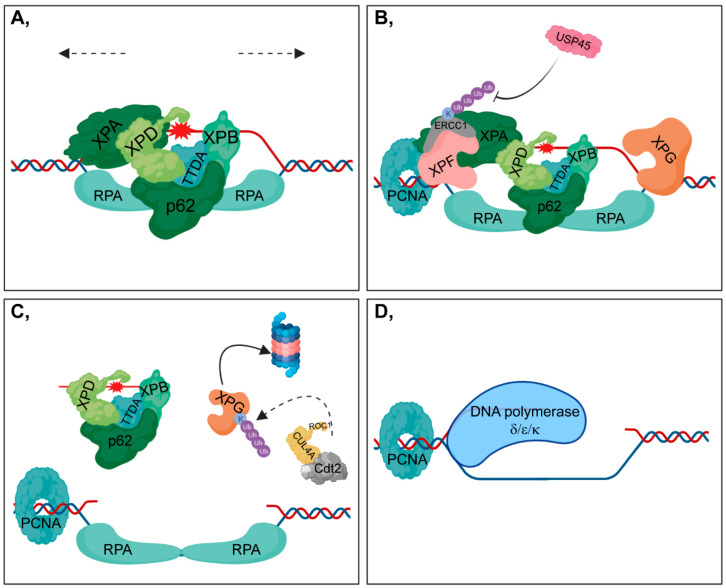
DNA damage verification and repair during NER (**A**) XPA (Xeroderma pigmentosum, complementation group A) recognizes modified nucleotides at the damaged strand and consequently facilitates the recruitment of TFIIH (including XPD (Xeroderma pigmentosum, complementation group D), XPB (Xeroderma pigmentosum, complementation group B), TTDA, and p62 subunits). Although the two helicase subunits of TFIIH, XPB and XPD, act as scaffold proteins during transcription, both have functional importance in DNA unwinding in NER (indicated with dashed arrows) [63,83]. After the partial opening of the DNA helix, RPA (Replication protein A) joins the complex, which then contributes to damage verification. XPA preferably binds to kinked and branched dsDNA (double-strand DNA) structures than to single-stranded DNA, while RPA can be observed only at ssDNA (single-strand DNA) regions [84]. Moreover, in the pre-incision bubble, XPA has been shown to be located on the 5’-side of the lesion [85]. (**B**) XPF-ERCC1 (Xeroderma pigmentosum, complementation group F - Excision Repair Cross-Complementation Group 1) catalyzes the 5’ incision, while XPG (Xeroderma pigmentosum, complementation group G) is responsible for the 3’ incision around the lesion. ERCC1 is polyubiquitylated at K33, which can be removed by USP45 (Ubiquitin specific peptidase 45). PCNA (Proliferating cell nuclear antigen) is loaded onto the 5’ end of DNA. PCNA interacts with XPA and XPF, stimulating their activity [86,87]. (**C**) The lesion-containing 22-30 nucleotide DNA region is excised from the DNA in complex with TFIIH, which is then slowly released from TFIIH and becomes bound by RPA or degraded by nucleases [88]. During the incision steps, XPG is simultaneously ubiquitylated by CRL4^Cdt2^ and is then degraded in the 26S proteasome. (**D**) DNA synthesis is catalyzed by DNA polymerase δ/ε/κ.

First, TFIIH binds to the site of DNA damage, presumably with the contribution of the ATPase activity of XPB (Xeroderma pigmentosum, complementation group B) (encoded by ERCC3) strengthened by the TTDA (also known as GTF2H5) subunit of TFIIH [61] (Figure 3A). Simultaneously, the CAK (CDK-activating kinase) subcomplex dissociates from TFIIH [62]. XPB and XPD (Xeroderma pigmentosum, complementation group D) (encoded by ERCC2) promote the unwinding of the DNA strand around the lesion through their 3’-5’ and 5’-3’ helicase activity, respectively [63,64] (Figure 3A). 

Next, XPA (Xeroderma pigmentosum, complementation group A) recognizes the damaged nucleotides and participates in the recruitment of further downstream repair factors to the DNA [65] (Figure 3A). However, XPA is tightly regulated by post-translational modifications. HERC2 (HECT Domain and RCC-Like Domain-Containing Protein 2)-mediated ubiquitylation of XPA can be attenuated through ATR (Ataxia Telangiectasia and RAD3-Related Protein)-mediated phosphorylation of XPA contributing to its stabilization [66]. Furthermore, PARylation of XPA impairs its DNA-binding affinity [67].

Following DNA unwinding, the single-strand DNA is coated and protected by RPA (Replication protein A), which help to position XPF-ERCC1 and XPG (encoded by ERCC5) endonucleases to the regions in the close vicinity of the lesion [68] (Figure 3A,B). XPF-ERCC1 heterodimer catalyzes the 5’ incision, while XPG is responsible for the 3’ incision of the lesion resulting in the removal of a 22-30 nucleotide region [69,70]. ERCC1 is polyubiquitylated at its C-terminal (HhH)_2_ (double helix–hairpin–helix) domain via K33 chains of ubiquitin through which it can heterodimerize with XPF [71,72]. However, XPF is not polyubiquitylated, and its protein level is significantly associated with that of ERCC1, suggesting that heterodimer formation is necessary for the stability of the complex [73,74]. USP45 (Ubiquitin specific peptidase 45) is essential for this process, because it participates in the removal of ubiquityl groups from ERCC1 [75] (Figure 3B). 

After the 5’ incision, PCNA (Proliferating cell nuclear antigen) is loaded onto the 5’ end of the DNA for gap filling through XPG [76] (Figure 3B). However, other evidence indicates that PCNA can be exclusively recruited when the damaged DNA region has been completely excised [77]. Furthermore, Cdt2 (Cell division cycle protein), one of the transiently bound subunits of the CRL4 complex, is involved in the ubiquitylation-mediated degradation of XPG in the presence of PCNA [78] (Figure 3C). This step is important in facilitating further gap filling during DNA synthesis. DNA polymerases δ, ε and κ are responsible for DNA synthesis at the damaged strand using the undamaged strand as a template [79,80] (Figure 3D). Moreover, DNA polymerase δ can also serve as a substrate of the CRL4^Cdt2^ complex [81]. In the last step of NER, XRCC1-Ligase III mediates gap filling throughout the cell cycle, and Ligase I participates in this process during the S phase [82].

## 3. Precise Coordination of Ubiquitin-Mediated Removal of RNAPII upon Transcription Blockage

Upon DNA damage, RNAPII becomes hyperphosphorylated to avoid initiation of a new transcription cycle until the damage sites are repaired [89]. As a ‘last resort’, when the damaged DNA cannot be repaired by TC-NER, S2P RNAPII has to be removed from the DNA through the ubiquitin-proteasome system to allow access for the NER factors [8]. Ubiquitylation of S2P RNAPII can be initiated when TC-NER is activated after the blockage of transcription elongation [90] (Figure 2B). In mammalian cells, CSA, BRCA1-BARD1, and NEDD4 ubiquitin ligases are essential in the mono- or polyubiquitylation of S2P RNAPII. If transcription arrest cannot be resolved, S2P RNAPII is polyubiquitylated by the Elongin A/B/C-Cul5-RBX2 complex, but it can act only in the presence of NEDD4 [6,91] (Figure 2B,F). In yeast, UBP2 and UBP3 mediate the deubiquitylation of S2P RNAPII [6]. UBP2 can trim K63-linked, while UBP3 catalyzes the removal of K48-linked polyubiquitin chains from S2P RNAPII, which, in turn, leads to its monoubiquitylation [8] (Figure 2F).

In case of serious DNA damage, several factors contribute to the blockage of transcription and the removal of S2P RNAPII from the damaged site. DNA-PK and WWP2 (WW domain containing E3 ubiquitin protein ligase 2) E3 ligase, which are involved in DSB repair, mediate transcription arrest upon DSB induction [92,93]. Following DNA-PK inhibition, S2P RNAPII can bypass the break site, suggesting that it is not the DNA-PK itself, but its activity, that is the key feature of this process [92]. Furthermore, one of the targets of DNA-PK, P53, interacts with RNAPII and might contribute to the removal of the S2P RNAPII upon Actinomycin D-induced transcription elongation blockage, and it facilitates the proteasome-mediated degradation of RNAPII at a transcribed unit [94].

The 26S proteasome can bind to actively transcribed gene regions at which RNAPII occupancy is high. This suggests that upon transcription arrest, the proteasome may degrade RNAPII at the site of damage [93,95]. As a first step of this process, the 19S subunit of the proteasome associates with RNAPII during transcription elongation. Upon transcription blockage, the 19S subunit, similar to a chaperone, takes part in the reassembly of the stalled transcription complex in a proteolysis-independent manner, thereby promoting the resumption of transcription elongation [96,97]. In contrast, the 20S subunit is involved in the degradation of the terminally stalled RNAPII [96].

## 4. Concluding Remarks and Future Perspectives

Preserving genome integrity is essential for normal cell physiology and is also necessary for maintaining the parental genetic information during replication. On the other hand, mutations in the genome can be beneficial for adaptation to environmental and evolutionary challenges. For this, dedicated balance is important during DNA repair. NER, which is specialized in repairing UV-induced damage, is a tightly regulated process, in which PTMs, including ubiquitylation, play a pivotal role. The proper coordination of the repair mechanism and the maintenance of genome integrity are ensured by NER factors, including ubiquitin ligase complexes.

In GG-NER, the main ubiquitin ligase complex is CRL4^DDB2^, which has opposite effects on its targets, although both are polyubiquitylated through K48 chains: (I) catalyzing the ubiquitylation of DDB2 results in its proteasomal-dependent degradation, while (II) ubiquitylating XPC, one of the initial repair proteins bound to the UV-damaged sites, leads to its more tenacious DNA-binding. Interestingly, K48-linked polyubiquitylation does not result in the proteasomal degradation of XPC, which needs to be clarified in the future. Since neither the E2 ubiquitin-conjugating enzyme involved in this process nor the ubiquitylated amino acid residue has been identified, both may explain this phenomenon. In contrast, RNF111 is responsible for the K63-linked polyubiquitylation of XPC, resulting in its removal from the damaged DNA to allow access for further NER factors. Moreover, CRL4^Cdt2^ catalyzes the polyubiquitylation of XPG participating in the downstream incision steps. 

During TC-NER, the proper balance between CRL4^CSA^ and UVSSA-USP7 has an indispensable effect on the access of downstream NER factors to the damaged region by mediating the ubiquitin-proteasomal degradation of CSB, in which process the BRCA1-BARD1 complex is also implicated. As a result of serious DNA damage, the stalled RNAPII must be entirely removed from the damaged region with the contribution of several ubiquitin ligases, including NEDD4, BRCA1-BARD1, Elongin A/B/C-Cul5-RBX2, and WWP2. In certain cases, ubiquitylation-linked signalization is crucial, because NER factors cannot bind to the damaged sites without the removal of the stalled S2P RNAPII.

In addition to ubiquitin ligases, DUBs also have a substantial function in the fine-tuning of NER. UBP12 and USP24 have been shown to contribute to the removal of K48-linked polyubiquitylation chains from DDB2 in fission yeast and in humans, respectively. USP51 is responsible for deubiquitylating the K13-15 residues of H2A, the exact role of which has not yet been clearly defined. XPC can be recycled by the removal of K48-linked polyubiquitin chains catalyzed by either USP7 or OTUD4. Furthermore, USP7 is essential for the ablation of K48-linked polyubiquitin chains from CSB. USP45 can remove the K33-linked polyubiquitin chains from ERCC1. In *Saccharomyces cerevisiae*, UBP2 and UBP3 are responsible for the removal of K63-linked and K48-linked polyubiquitin chains of RNAPII, respectively. Accordingly, DUBs can provide additional levels for the fine-tuning of DDR. Genes encoding DUB enzymes affected by mutations have recently been linked to various diseases, including cancer, and have been identified as promising drug targets for eliminating tumorous malformations. Unlike E3 ligases discussed in this review, DUBs are promising candidates for small-molecule drug targets for developing novel cancer therapeutics.

As discussed above, maintaining the proper balance between E3 ubiquitin ligases and DUBs is indispensable for the stringent coordination of NER pathways. In response to UV irradiation, the precise function of these factors ensures the preservation of genome integrity in a coordinated spatiotemporal manner, thereby significantly contributing to the prevention of cancerous malformations. We have already shown that SerpinB2 can play a role in the regulation of the NER pathway through the XPB protein as well as the ubiquitin network, and this function is altered in tumor cells [98]. Hence, being aware of any malfunction of even one of these factors can contribute to the better understanding the molecular background of the tumor. Consequently, applying either agonists or antagonists of the maleficent molecule can be beneficial in personalized tumor therapy.
